# Alveolar Ridge Preservation Using a Novel Synthetic Grafting Material: A Case with Two-Year Follow-Up

**DOI:** 10.1155/2018/6412806

**Published:** 2018-02-01

**Authors:** Peter Fairbairn, Minas Leventis, Chas Mangham, Robert Horowitz

**Affiliations:** ^1^Department of Periodontology and Implant Dentistry, School of Dentistry, University of Detroit Mercy, 2700 Martin Luther King Jr. Boulevard, Detroit, MI 48208, USA; ^2^Department of Oral and Maxillofacial Surgery, Dental School, National and Kapodistrian University of Athens, 2 Thivon Street, Goudi, Athens 115 27, Greece; ^3^Manchester Molecular Pathology Innovation Centre, The University of Manchester, Nelson Street, Manchester M13 9NQ, UK; ^4^Departments of Periodontics, Implant Dentistry, and Oral Surgery, New York University College of Dentistry, 345 E. 24th Street, New York, NY 10010, USA

## Abstract

This case report highlights the use of a novel in situ hardening synthetic (alloplastic), resorbable, bone grafting material composed of beta tricalcium phosphate and calcium sulfate, for alveolar ridge preservation. A 35-year-old female patient was referred by her general dentist for extraction of the mandibular right first molar and rehabilitation of the site with a dental implant. The nonrestorable tooth was “atraumatically” extracted without raising a flap, and the socket was immediately grafted with the synthetic biomaterial and covered with a hemostatic fleece. No membrane was used, and the site was left uncovered without obtaining primary closure, in order to heal by secondary intention. After 12 weeks, the architecture of the ridge was preserved, and clinical observation revealed excellent soft tissue healing without loss of attached gingiva. At reentry for placement of the implant, a bone core biopsy was obtained, and primary implant stability was measured by final seating torque and resonance frequency analysis. Histological analysis revealed pronounced bone regeneration while high levels of primary implant stability were recorded. The implant was successfully loaded 12 weeks after placement. Clinical and radiological follow-up examination at two years revealed stable and successful results regarding biological, functional, and esthetic parameters.

## 1. Introduction

Clinical and experimental studies have shown that grafting the postextraction sockets at the time of tooth extraction with a bone grafting material constitutes a predictable and reliable way to limit the resorption of the alveolar ridge [1–3]. Such alveolar ridge preservation measures involve the use of a wide variety of bone substitutes, barrier membranes, and biologically active materials, and many different surgical techniques and protocols have been proposed [4–6].

According to Yip et al. [7], the ideal grafting material should have specific attributes. It should be osteoconductive, osteoinductive, and biocompatible. It is important to be totally replaced by host bone having an appropriate resorption time in relation to new bone formation. Moreover, it should be able to maintain the volume stability of the augmented site, have satisfactory mechanical properties, and have no risk of disease transmission.

Allografts, xenografts, and synthetic particulate materials, with or without a membrane, have been extensively used and documented, showing adequate results in the preservation of the ridge dimensions [5]. It is important that these bone substitutes vary in terms of origin, composition and biological mechanism of function regarding graft resorption and new bone formation, each having their own advantages and disadvantages [8, 9].

Alloplasts represent a group of synthetic osteoconductive, biocompatible bone substitutes that are free of any risk of transmitting infections or diseases by themselves, and their availability is unlimited [10–12]. One of the most promising groups of synthetic bone substitutes is calcium phosphate ceramics, and among them beta tricalcium phosphate (*β*-TCP) is commonly used [13–15]. Apart from being osteoconductive, there is strong experimental evidence that calcium phosphates also have osteoinductive properties. Although the underlying mechanism remains largely unknown, it has been shown that these alloplastic materials can stimulate osteogenic differentiation of stem cells in vitro and bone induction in vivo [16, 17].

The ability of the bacteriostatic calcium sulfate (CS) to set and hence be stable is well documented. Adding CS to *β*-TCP produces an in situ hardening grafting material that binds directly to the host bone, maintains the space and shape of the grafted site, and acts as a stable scaffold [18–23]. The improved stability throughout the graft material seems to further improve the quality of the bone that will be regenerated due to reduced micromotion of the material, which may lead to mesenchymal differentiation to fibroblasts instead of osteoblasts. It is known that micromovements between bone and any implanted grafted material prevent bone formation, resulting in the development of fibrous tissue [24, 25]. Moreover, the CS element creates a nanoporous cell occlusive membrane that may prevent the early stage invasion of unwanted soft tissue cells into the graft [26, 27].

Both CS and *β*-TCP are fully resorbable materials leading to the regeneration of high-quality vital host bone without the long-term presence of residual graft particles. The CS element will resorb over a 3–6-week period, depending on patient physiology, thus increasing porosity in the *β*-TCP scaffold for improved vascular ingrowth and angiogenesis, while the *β*-TCP element will resorb by hydrolysis and cellular resorption over a period of 9–16 months, again dependent on host physiology [13].

The purpose of this report is to present a case that highlights the clinical, radiological, and histological outcomes of socket grafting with an in situ hardening *β*-TCP/CS synthetic bone substitute following a minimally invasive procedure.

## 2. Case Presentation

**Figure 1:**
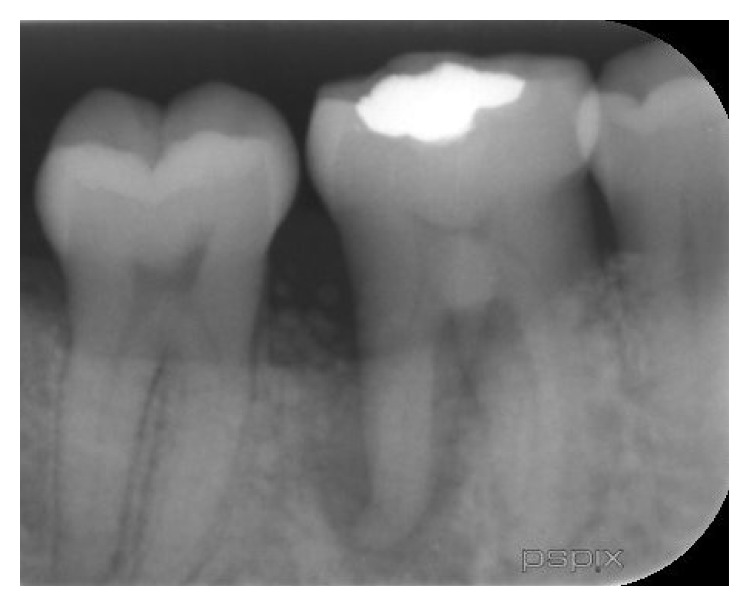
Preoperative periapical X-ray.

A 35-year-old female patient, nonsmoker, with noncontributory medical history, presented with a nonconservable mandibular right first molar due to extensive caries and periapical pathology (Figure 1). After thorough clinical and radiological examination, a delayed implant placement treatment plan was decided, consisting of extraction of the failing tooth with simultaneous socket grafting and implant placement after a 12-week healing period.

**Figure 2:**
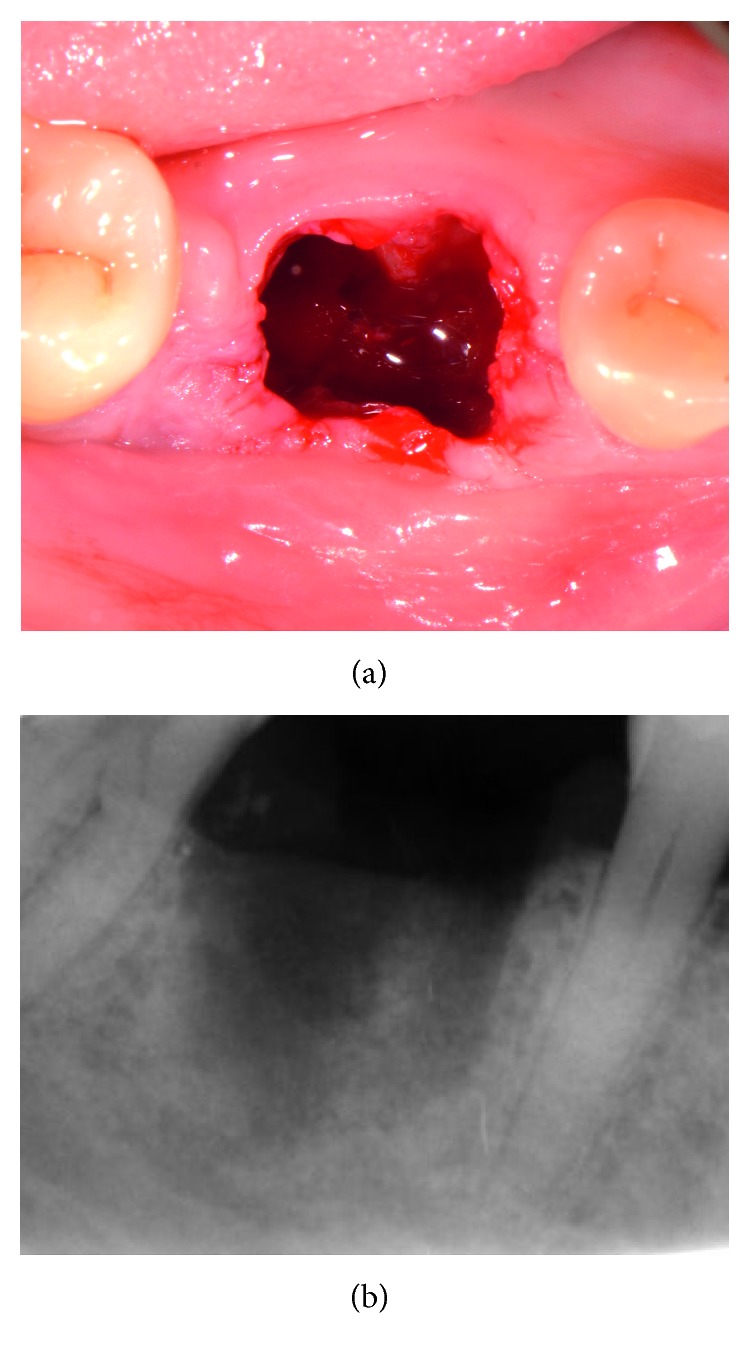
Clinical view and periapical X-ray of the site immediately after the “atraumatic” flapless extraction. The septal bone is completely missing, and the buccal bone was defective. Note the shortage of buccal keratinized soft tissue.

Tooth extraction was performed under local anesthesia without flap elevation. In order to minimize surgical trauma, the tooth was sectioned with a Lindemann burr (Komet Inc., Lemgo, Germany) under copious irrigation with sterile saline, and each root was independently mobilized and carefully luxated using periotomes and elevators. Attention was given not to injure the surrounding soft and hard tissues, especially in the buccal aspect. After extraction, the socket was thoroughly debrided from granulation tissue, using bone curettes, and rinsed with sterile saline. A periodontal probe was then utilized to explore the site which revealed that the septal bone and the buccal bone wall were completely missing (Figure 2).

**Figure 3:**
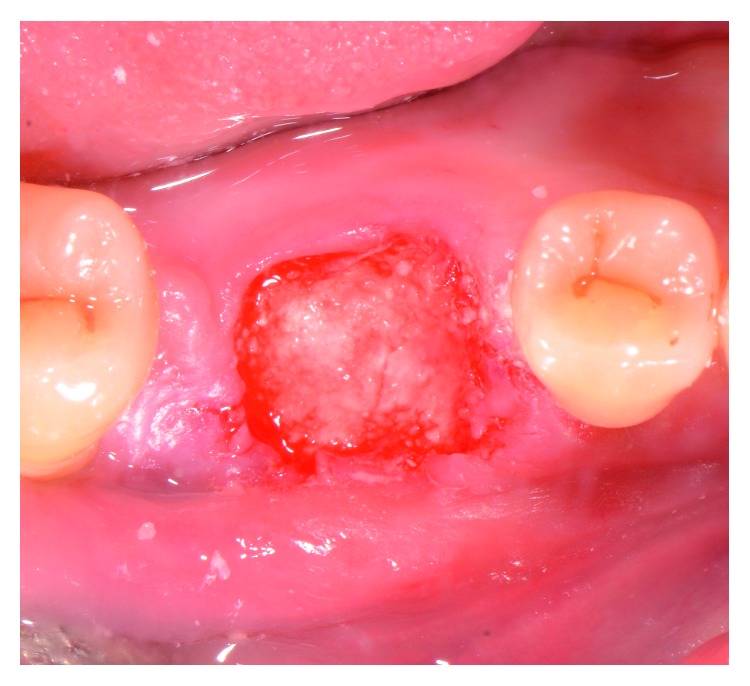
Grafting of the site with the in situ hardening synthetic bone substitute. Attention was given not to overfill the socket.

**Figure 4:**
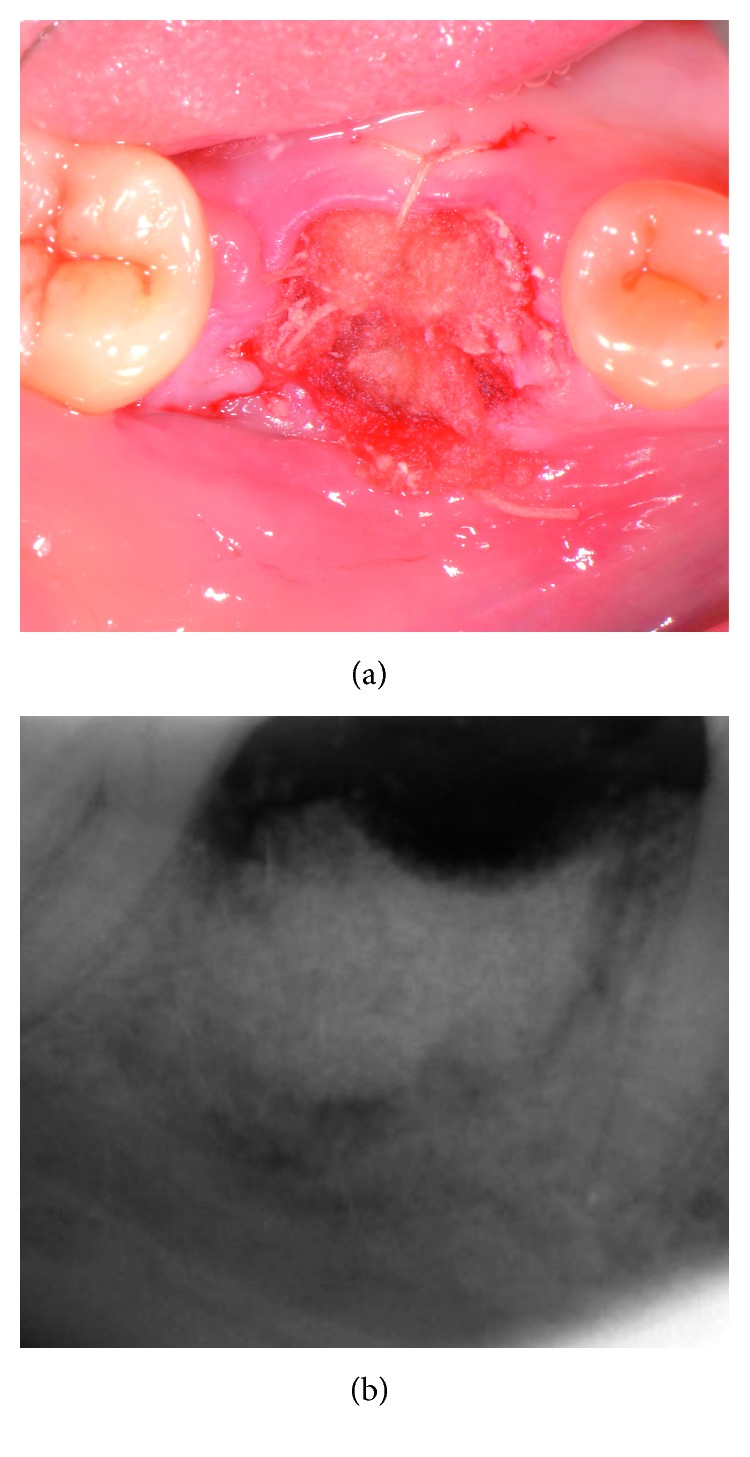
Clinical view and periapical X-ray of the grafted site.

A fully resorbable alloplastic in situ hardening bone substitute (EthOss^®^, Ethoss Regeneration Ltd., Silsden, UK) was used to graft the site. The material consists of *β*-TCP (65%) and CS (35%), preloaded in a plastic sterile syringe. In accordance with the manufacturer's instructions prior to injecting the material into the socket, the particles of the biomaterial were mixed in the syringe with sterile saline. After application of the graft into the postextraction site, a bone plunger was used to condense the moldable graft particles, in order to occupy all the volume of the socket up to the level of the surrounding host bone (Figure 3). Attention was given not to overfill the socket as this could result in subsequent sequestration of the exposed coronal particles or displacement of the entire graft mass after mechanical irritation during the first phases of healing. A saline-wet gauze was used to further compact the graft particles and accelerate the in situ hardening of the CS element of the graft. As a result, after a few minutes, the alloplastic bone substitute formed a stable, porous scaffold for the host osseous regeneration. The site was then covered with a hemostatic dressing material (Jason^®^ Collagen Fleece, Botiss Biomaterials GmbH, Germany), and a cross-mattress tension-free 5/0 suture (Vicryl^®^, Ethicon, Johnson & Johnson, Somerville, NJ, USA) was placed over to achieve soft tissue stability (Figure 4). The site was left uncovered without obtaining primary closure in order to heal by secondary intention. The patient did not wear any prosthesis during the healing period. Antibiotic therapy consisting of 500 mg amoxicillin every 8 hours for 5 days, and a 0.2% chlorhexidine mouthwash for 7 days were prescribed. The suture was removed 1 week postoperatively.

**Figure 5:**
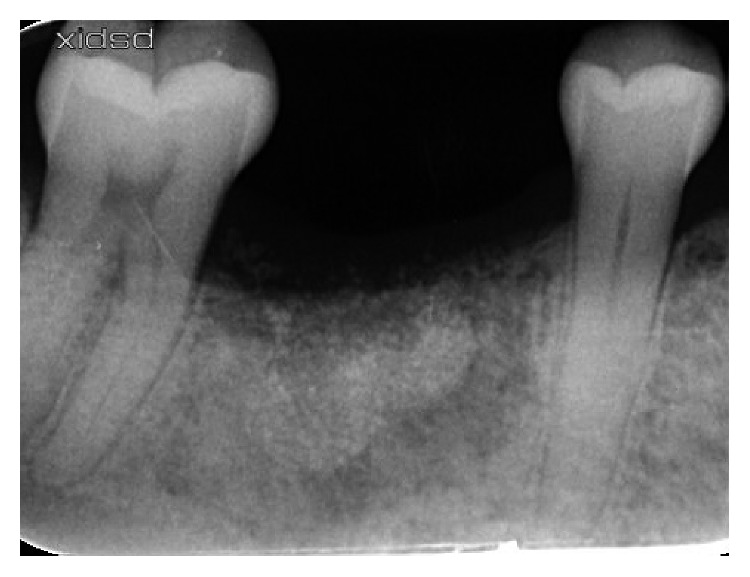
Periapical X-ray at 12 weeks showing that the site is occupied by newly formed hard tissue.

**Figure 6:**
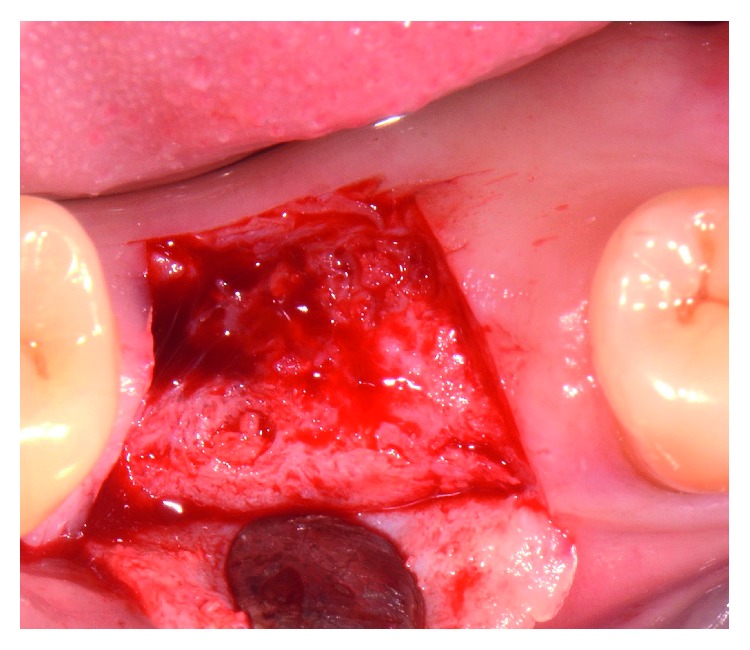
Clinical reentry after 12 weeks of healing. The site is occupied by newly formed hard tissue.

**Figure 7:**
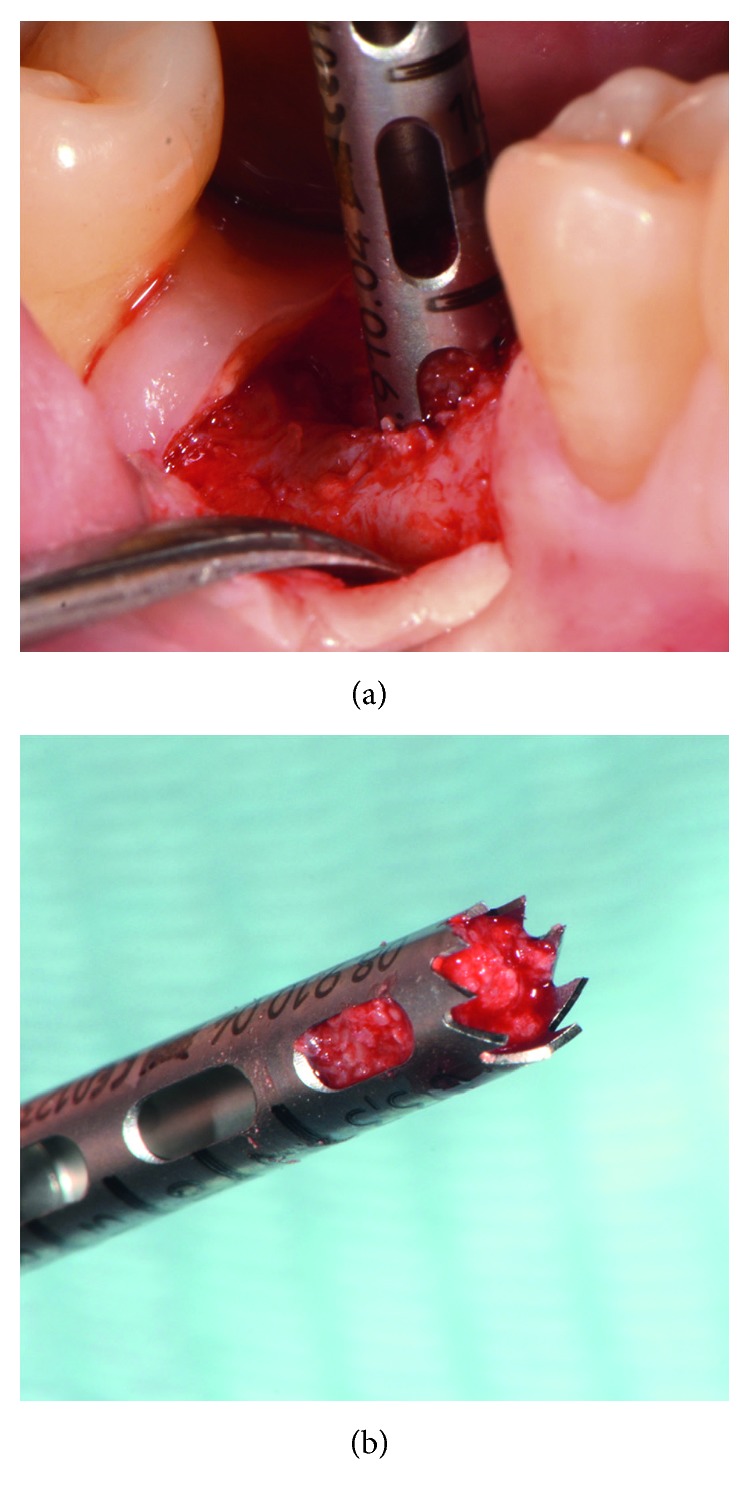
Trephine bone biopsy taken at the center of the regenerated site.

**Figure 8:**
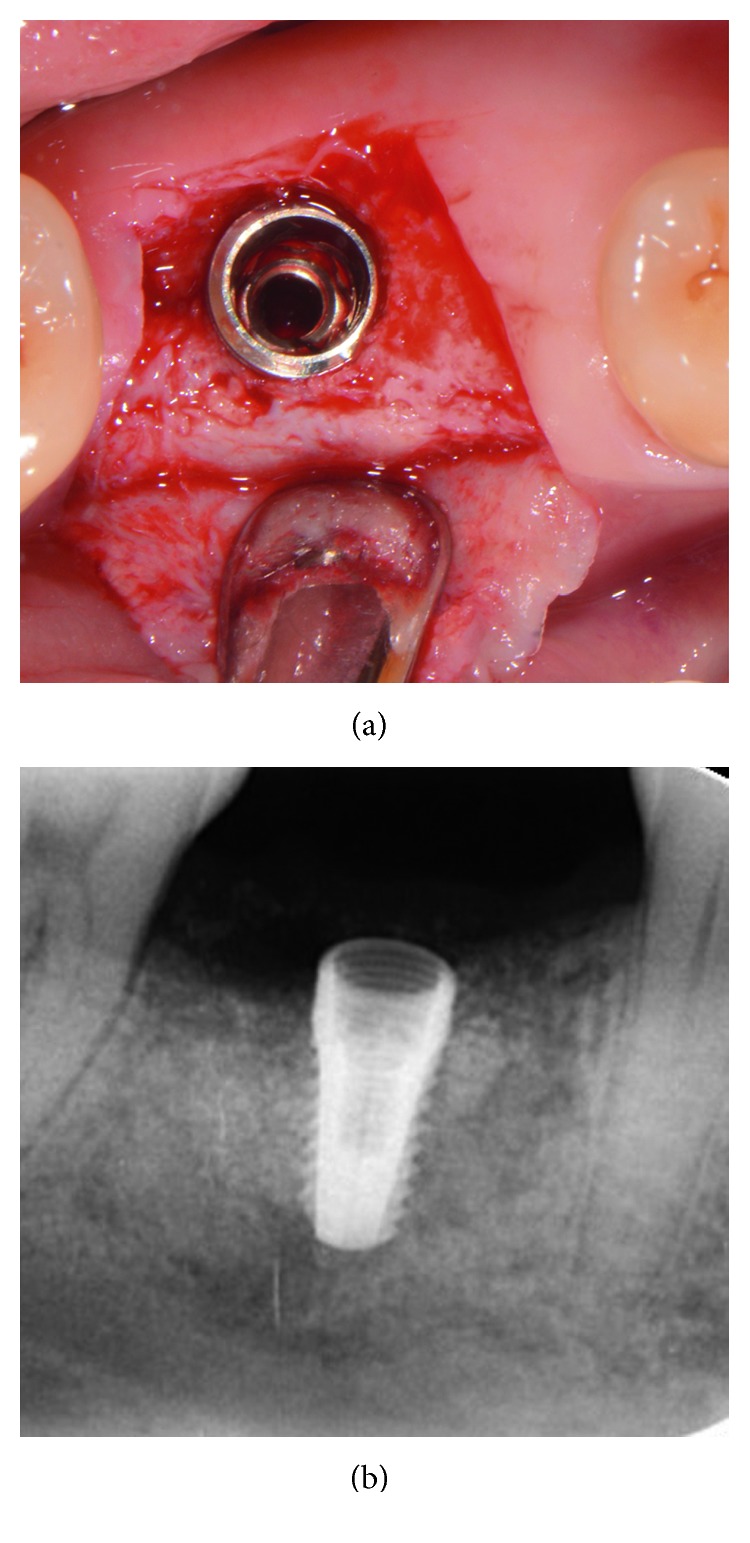
Implant placement at the optimal positioning.

The postoperative healing was uneventful, and the site was gradually covered by newly formed soft tissue with no loss of bone graft particles. After 12 weeks, the area was completely covered with newly formed keratinized epithelium, while the volume and architecture of the ridge were adequately preserved. A periapical X-ray at this point in time showed the consolidation of the grafting material, resulting in bone regeneration at the site (Figure 5). A site-specific full thickness flap was elevated revealing that the grafted area was filled with regenerated hard tissue (Figure 6). Prior to implant placement, a bone core biopsy was taken (Figure 7) with a depth of 7 mm from the center of the site using a trephine drill with a diameter of 2.3 mm (Komet Inc., Lemgo, Germany). Following the harvesting of the bone sample, the preparation of the bony bed was completed at the same site and a tapered implant (Dio Co., Busan, Korea), of 4.5 mm in diameter and 10 mm in length, was then placed at the optimal position (Figure 8). Immediately after implant placement, the final seating torque was recorded using the manufacturer's hand ratchet (Dio Co., Busan, Korea). The ISQ was also measured by resonance frequency analysis (Osstell ISQ™, Göteborg, Sweden) showing excellent initial stability (35 Ncm and 69/70 resp.). The cover screw was placed, and the mucoperiosteal flap was repositioned and closed, without tension, using interrupted resorbable 4-0 sutures (Vicryl, Ethicon, Johnson & Johnson, Somerville, NJ, USA).

**Figure 9:**
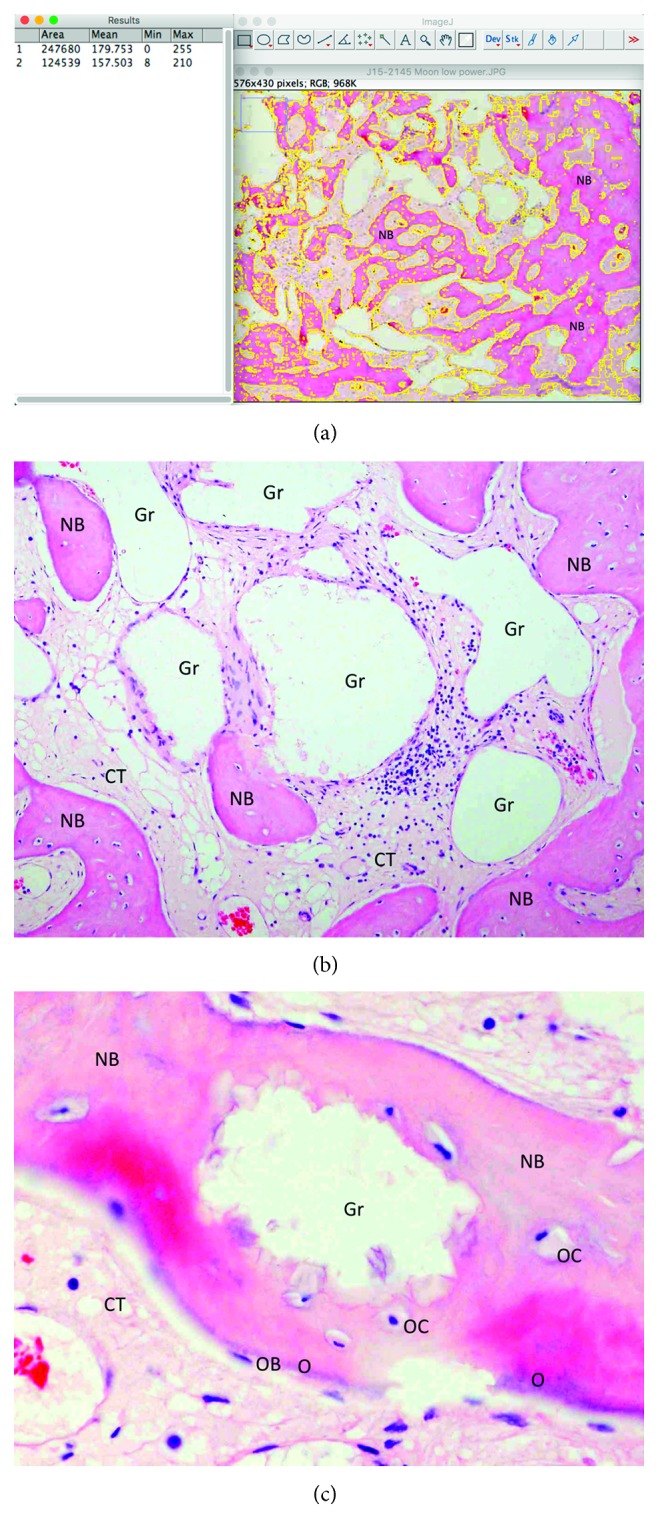
Histological sections of bone core biopsy harvested from the regenerated site using a trephine burr. (a) Overview image of the coronal-apical cut through the entire core biopsy showing new bone (NB) formation in the extraction socket. The new bone is marked, and its surface was calculated in accordance to the overall surface of the specimen, revealing a 50.28% percentage of new bone (H&E stain, original magnification ×20). (b) EthOss particles (Gr) are embedded in well-perfused connective tissue (CT) and newly formed bone (NB) (H&E stain, original magnification ×200). (c) High magnification showing new bone (NB) in contact with a graft particle (Gr). Osteocytes (OC) can be detected inside the new bone and osteoblasts (OB) forming osteoid (O) on its outer surface (H&E stain, original magnification ×400).

The bone specimen was fixed in 10% formalin for 2 days and subsequently decalcified in bone decalcification solution for 14 days. After routine tissue processing the entire core was embedded into paraffin wax, orientated for longitudinal sectioning. 4 *μ*m-thick tissue sections were cut and stained with hematoxylin and eosin (H&E) for light microscopical examination. Histologically, the analyzed biopsy contained newly formed bone, residual grafting material, and vascularized uninflamed connective tissue. No necrosis or foreign body reactions were detected. The graft particles were surrounded by or in contact with trabecular bone, while active osteoblasts forming osteoid and new woven bone could be identified, demonstrating persistent osteogenesis (Figure 9). Histomorphometric analysis was performed “blind” by one independent observer using the ImageJ imaging analysis software (NIH Image, National Institutes of Health, Maryland, USA). The reference area was the entire area in the biopsy. Histomorphometric analysis revealed that after 12 weeks of healing, the grafted site was occupied by 50.28% of new bone, 12.27% of residual grafting material, and 37.45% of connective tissue.

**Figure 10:**
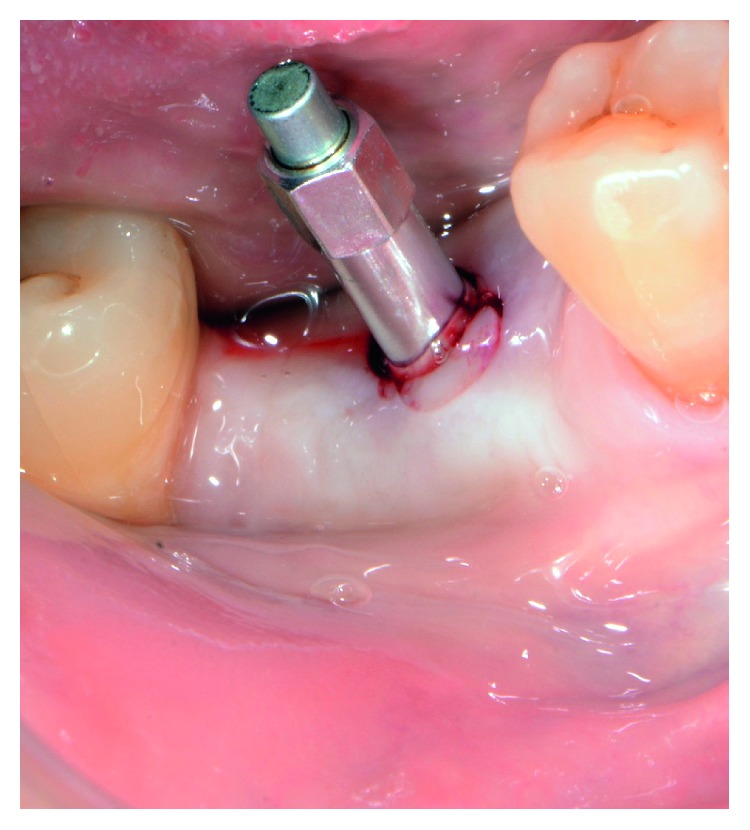
ISQ measurement at uncovering of the implant.

**Figure 11:**
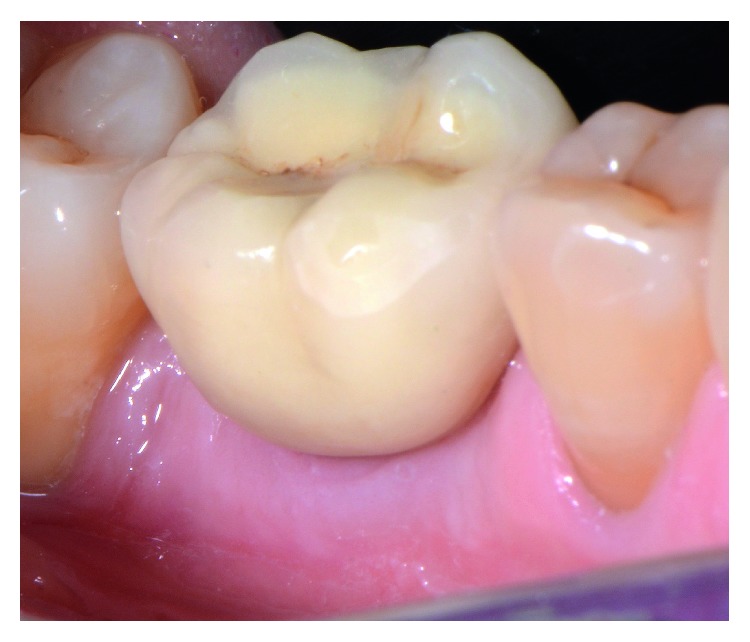
Clinical view of the restoration immediately after fitting the restoration.

After allowing 10 weeks for osseointegration, the implant was accessed using a tissue punch, and a higher ISQ (77) was measured (Figure 10). Two weeks later, the implant was restored with the final stock titanium abutment (Dio Co., Busan, Korea) and a cement-retained lithium-disilicate glass-ceramic crown (PS e.max Press; Ivoclar Vivadent AG, Schaan, Liechtenstein), achieving pleasant clinical and radiological results (Figure 11).

**Figure 12:**
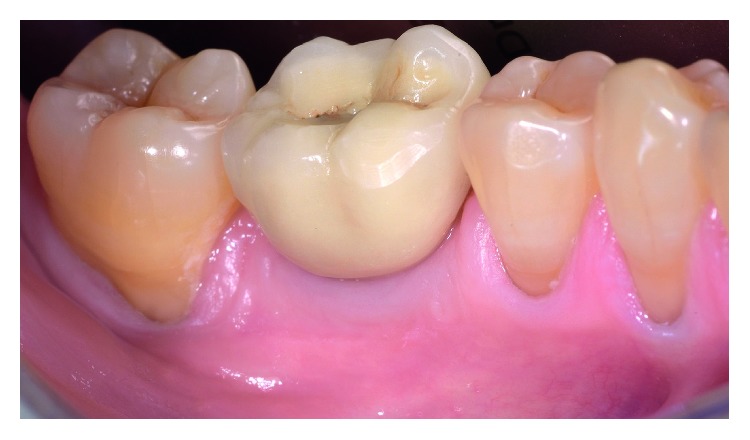
Clinical view two years after loading the implant.

**Figure 13:**
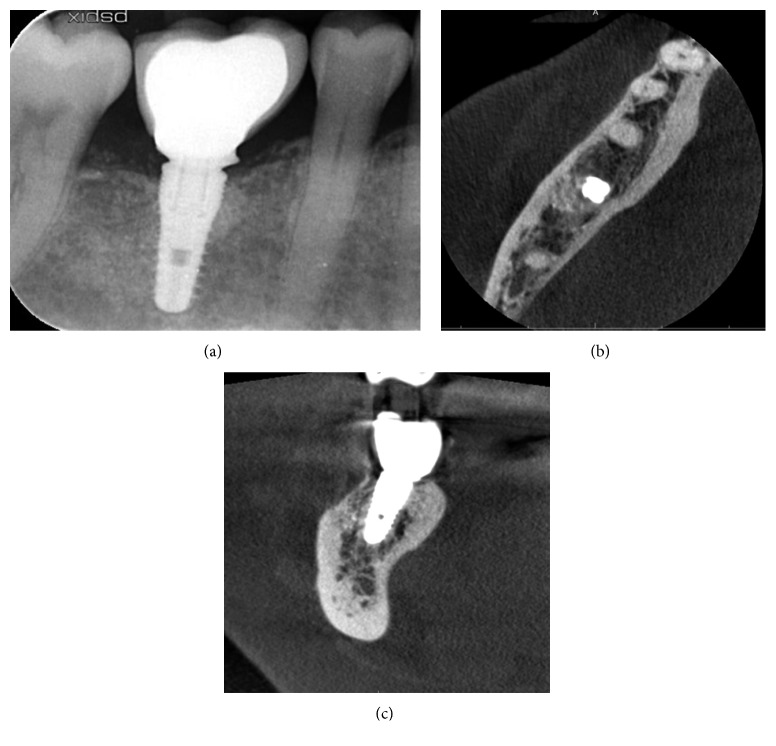
Radiological examination two years after loading of the implant: periapical X-ray and axial and coronal planes of the CBCT showing the preservation of the dimensions of the regenerated bone.

Follow-up clinical examination two years after loading revealed stable peri-implant keratinized soft tissues with excellent preservation of the volume and architecture of the ridge (Figure 12). A periapical X-ray and CBCT showed further functional remodeling of the bone around the loaded implant with no radiological findings of residual biomaterial (Figure 13).

## 3. Discussion

In this case, a minimally invasive protocol was followed. Extraction and socket grafting were performed without raising a flap, and the augmented site was not covered with a barrier membrane nor a flap. This approach was selected in order to minimize patient morbidity, surgical time, and cost, but mostly in an attempt not to displace the mucogingival junction and to allow for the spontaneous formation of new soft tissue over the postextraction grafted site, as described in similar studies using self-hardening synthetic bone grafting materials [11, 28]. The biomechanical stability of the *β*-TCP/CS graft used in the presented case allowed the site to heal gradually by secondary intention, without loss of the exposed biomaterial in the oral environment. Elevating and advancing a full thickness flap for covering the socket will protect the grafting material. However, it is shown that achieving primary closure does not present beneficial effects on preserving the ridge width. In addition, patients experience more discomfort, and the distortion of the vestibule and the coronal displacement of the buccal keratinized gingiva may lead to esthetic problems, alter the soft tissue profile of the site, and influence in a negative way the health status of the supporting tissues around dental implants [4, 29].

It is without doubt that bone quality is of paramount importance in successful implant therapy. According to Horváth et al. [2], it is doubtful whether an alveolar ridge preservation method should be claimed successful, if it only preserves the external contour of the alveolar ridge, but the newly formed hard tissue is of inferior quality and quantity (percentage of matured trabecular bone) to what is spontaneously achieved following a tooth extraction. Contemporary literature reports conflicting results with the use of the widely used xenografts, with changes in the percentage of vital bone ranging from −22% (decrease) to 9.8% (increase), while considerable residual hydroxyapatite and xenogenic particles (15% to 36%) remained at a mean of 5.6 months after socket grafting procedures [9]. Although it remains unknown whether these changes in bone quality will affect implant success and peri-implant tissue stability in the long term, there is a concern that firstly the long-term, presence of residual nonresorbable or slowly resorbable graft particles might interfere with normal bone healing and remodeling, secondly it may reduce the bone-to-implant contacts, and thirdly it can have a negative effect on the overall quality and architecture of the bone that surrounds implants. In a recent systematic review of randomized controlled clinical trials analyzing the outcomes of flapless socket grafting, Jambhekar et al. [30] reported that, after a minimum healing period of 12 weeks, sockets filled with synthetic biomaterials had the maximum amount of vital bone (45.53%) and the least amount of remnant graft material (13.67%) compared to xenografts and allografts. The results of the present case are in accordance with the above findings as histomorphometry revealed 50.28% of new bone and 12.27% of residual graft 12 weeks after a flapless socket grafting procedure.

Augmenting the bone around implants using fully resorbable grafting materials like *β*-TCP and CS may raise concerns regarding the long-term volume stability of the site. However, the placement of the implant at 12 weeks will increase the metabolic activity of the regenerated bone, while the subsequent loading of the implant will trigger the remodeling, and gradually enhance the density of the surrounding hard tissues [31, 32]. Assuming that the newly formed hard tissue around the implants is high-quality vital bone with low content or no residual graft particles at all, it might be able to adapt successfully to the placement and loading of the implant and thus maintain its dimensions in a functional way. In the present case where resorbable synthetic materials were used, the ridge did not collapse and retained adequately the architecture and the volume of the hard tissues two years after loading of the implant.

Overall, bone quality is important and might influence in several ways the outcome of implant therapy [9]. However, clinicians should be aware that the remodeling of the regenerated bone around implants might not be affected only by the nature and presence of residual graft particles, but also by other factors like optimal 3D positioning of the implant, type of implant (bone/tissue level), opposing dentition, and occlusal forces, while success of socket grafting can also be influenced by many other parameters like healing time, smoking status, local and systemic factors, socket location, and surgical technique.

## 4. Conclusions

In the presented case, a novel synthetic resorbable grafting material composed of *β*-TCP and CS was utilized for alveolar ridge preservation, resulting in pronounced regeneration of high-quality bone capable to support implant placement after a 12-week healing period. In parallel, placing the implant 12 weeks after extraction and socket grafting, and subsequent loading of the implant at 12 weeks resulted in a functional preservation of the volume and dimensions of the site, as shown clinically and radiologically two years after loading in this case. The mechanical stability of self-hardening synthetic biomaterials may also enable clinicians to utilize minimally invasive flapless procedures without primary wound closure for socket grafting that reduce the patient's morbidity, while preserving the attached keratinized gingiva and allowing for further production of newly formed keratinized soft tissue. Additional studies, including larger samples, comparison of different materials, quantitative measurements of the ridge dimensional changes, and inclusion of different sites, like the anterior region, that might show more pronounced changes, are needed in order to confirm and supplement the present findings.
